# Lantibiotic production is a burden for the producing staphylococci

**DOI:** 10.1038/s41598-018-25935-2

**Published:** 2018-05-10

**Authors:** Patrick Ebner, Sebastian Reichert, Arif Luqman, Bernhard Krismer, Peter Popella, Friedrich Götz

**Affiliations:** 1 Microbial Genetics, Interfaculty Institute of Microbiology and Infection Medicine (IMIT), University of Tübingen, Auf der Morgenstelle 28, 72076 Tübingen, Germany; 2 Infection Biology, Interfaculty Institute of Microbiology and Infection Medicine (IMIT), University of Tübingen, Auf der Morgenstelle 28, 72076 Tübingen, Germany

## Abstract

Lantibiotics are antimicrobial peptides that contain non-proteinogenic amino acids lanthionine and 3-methyllanthionine and are produced by Gram-positive bacteria. Here we addressed the pros and cons of lantibiotic production for its producing strains. Two staphylococcal strains, *S. gallinarum* Tü3928 and *S. epidermidis* Tü3298 producing gallidermin and epidermin respectively were selected. In each of these parental strains, the structural genes *gdmA* and *epiA* were deleted; all the other biosynthetic genes including the immunity genes were left intact. Comparative analysis of the lantibiotic-producing strains with their non-producing mutants revealed that lantibiotic production is a burden for the cells. The production affected growth, caused release of ATP, lipids and increased the excretion of cytoplasmic proteins (ECP). The epidermin and gallidermin immunity genes were insufficient to protect the cells from their own product. Co-cultivation studies showed that the Δ*gdmA* mutant has an advantage over the parental strain; the latter was outcompeted. On the one hand, the production of staphylococcal lantibiotics is beneficial by suppressing competitors, but on the other hand they impose a burden on the producing-strains when they accumulate in higher amounts. Our observations explain why antibiotic-producing strains occur as a minority on our skin and other ecological niches, but retain corresponding antibiotic resistance.

## Introduction

Some Gram-positive bacterial species produce lantibiotics, which are lanthionine containing antimicrobial peptides with a length of usually 19 to 34 amino acids^1,2^. Lantibiotics such as gallidermin (Gdm), epidermin (Epi), or nisin are ribosomally synthesized in an inactive pre-form, the pro-lantibiotics^3^. The biosynthetic machineries are usually organized as gene-clusters comprising up to 11 genes. In *S. epidermidis*, and most likely also in *S. gallinarum* these genes are localized on an extrachromosomal plasmid^4^. They are organized in transcription units according to their function: immunity (*epi/gdmFEG*), export (*epi/gdmHT*), the short structural gene (*epiA/gdmA*) followed by the modification genes (*epi/gdmBCD*) and in opposite orientation, the regulatory (*epiQ/gdmQ*) and the protease genes (*epiP/gdmP*)^4^. The three enzymes LanB, LanC and LanD (*Epi/GdmBCD*) modify Gdm and Epi post-translationally^4^. LanB is catalyzing the dehydration of serine and threonine to dehydroalanine and dehydrobutyrine residues^5^, LanC forms the lanthionine ring^6^, and LanD decarboxylates the C-terminal end^7^. The pre-lantibiotics (composed of the pre-sequence and the modified core peptide) are secreted in an inactive form by LanHT (EpiHT/GdmHT) and are subsequently processed by the extracellular protease to the active lantibiotic (Epi/GdmP)^8^. *S. epidermidis* contains two frameshifts in *epiT*, which causes a decreased epidermin production. Interestingly, the translocation defect can be complemented by the homolog *gdmT* gene of the gallidermin gene cluster, which has an intact sequence^9^.

In the processed and active form, lantibiotics such as Epi, Gdm and nisin inhibit the cell wall biosynthesis by binding to Lipid II^10–12^. Self-defense of *S. gallinarum* and *S. epidermidis* is accomplished by LanFEG, of which most likely constitutes an ABC transporter that acts as an efflux pump^13,14^. For nisin, another immunity mechanism, the lipoprotein NisI binds and sequesters nisin in addition to the action of the NisFEG proteins^15^. NAI-107, a more recently discovered lantibiotic produced by Microbispora ATCC PTA-5024, possesses in total three immunity mechanisms: the MlbYZ ABC-transporter, the accessory membrane protein MlbJ, and the lipoprotein MibQ that is unrelated to LanI but also thought to bind the cognate lantibiotic^16^.

The self-defense of the lantibiotic producers is based on an immunity mechanism rather than classical resistance mechanism such as for vancomycin^17^, beta-lactams, or methicillin^18^. Therefore, lantibiotic production could be a burden to the producing- strains. For instance, the production of tropodithietic acid (TDA) by *Phaeobacter inhibens* causes a growth deficiency of the wildtype compared to the strain lacking the biosynthesis genes. Interestingly, inactivation of single biosynthetic gene caused a similar effect as the deletion of the entire gene cluster^19^. TDA caused partial destruction of the proton gradient that leads to a higher energetic demand.

In this study we show that the production of the two lantibiotics, epidermin and gallidermin, imposes a burden on the producing-strains. Mutants in which the structural gene has been deleted not only had a growth advantage but did not show the signs of membrane damage caused by the lantibiotics.

## Results

### Production of Gdm and Epi limits growth

**Figure 1:**
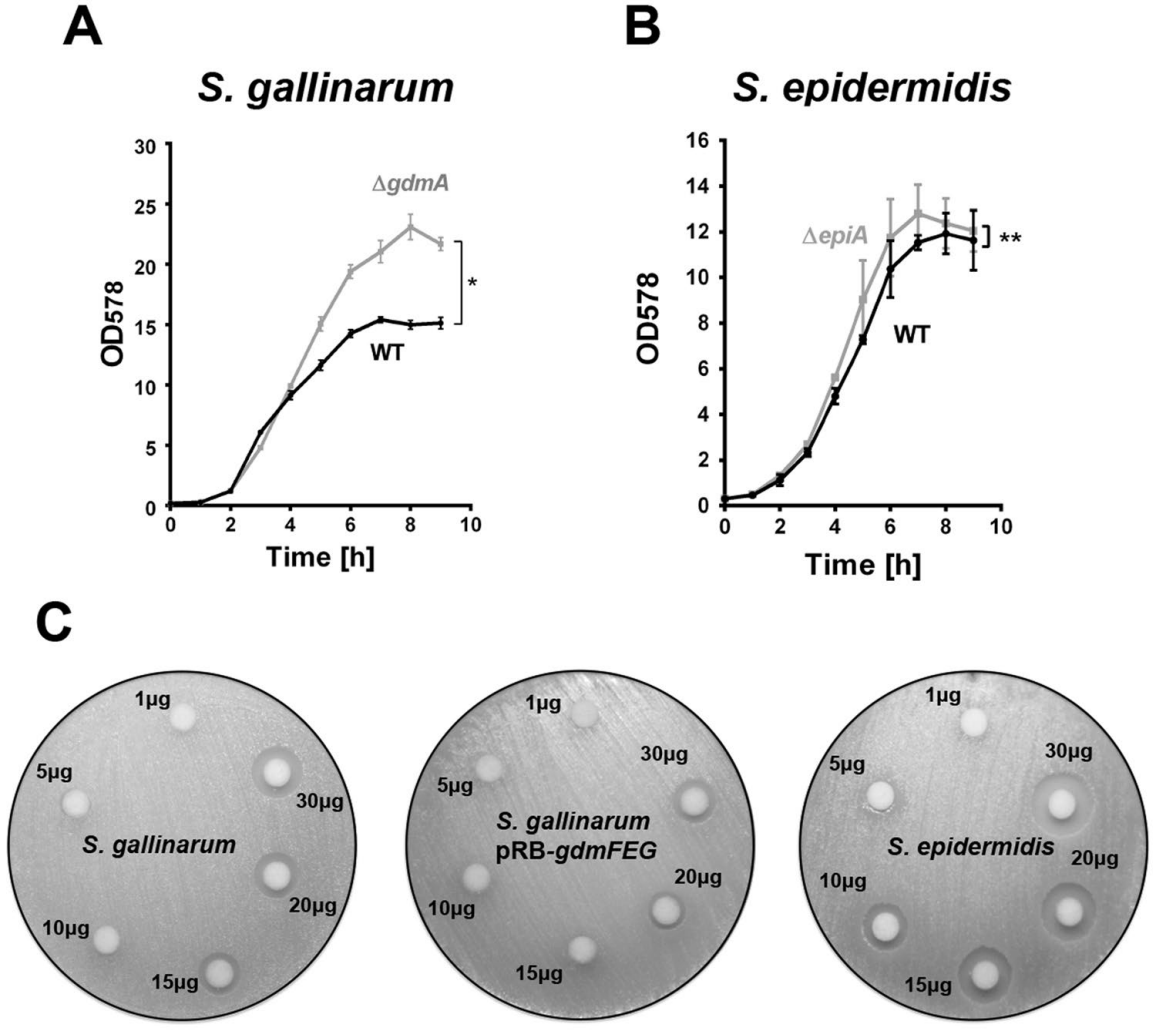
Growth of wild type strains with their lantibiotic-deficient mutants and Gdm-susceptibility comparison of the *S. gallinarum* and *S. epidermidis*. (**A**) *S. gallinarum* and its *gdmA* deletion mutant. (**B**) *S. epidermidis* and its *epiA* deletion mutant. Representative data from two independent experiments are shown. For all graphs, each data point represents the mean value ± SD *p < 0.05, **p < 0.01 by unpaired t-test comparing the parent strain with its corresponding *epiA/gdmA* mutants. (**C**) Agar-diffusion test with *S. gallinarum* (left), *S. gallinarum* pRB-*gdmFEG* (middle) and *S. epidermidis* (right) as reporter strain using increasing concentrations (1, 5, 10, 15, 20, 30 μg) of Gdm.

In order to see whether the production of Gdm and Epi affects growth of the producing-strains, we compared growth of the *gdmA* and *epiA* deletion mutants in *S. gallinarum* Tü3928 and *S. epidermidis* Tü3298 respectively with its corresponding parental strains. Each of the mutants grew significantly higher than its parental strains. (Fig. 1A,B). The generation time of *S. gallinarum* parent and its *gdmA* mutant was similar, 27.6 *vs* 29.6 min; while in *S. epidermidis* parent and its *epiA* mutant it was 49.0 *vs* 40.9 min. Although the generation time deviates to some extent, it does not affect the significantly higher OD obtained with both mutant strains. Moreover, in agar diffusion tests with increasing concentrations of Gdm, both *S. gallinarum* and *S. epidermidis* were inhibited in growth; the only difference was that *S. epidermidis* was more sensitive than *S. gallinarum*. We also investigated whether over-expressing the plasmid-encoded immunity genes *gdmFEG* in *S. gallinarum* renders the strain more resistant to Gdm. Indeed, *S. gallinarum* (pRB-*gdmFEG*) tolerated higher concentrations of Gdm (Fig. 1C).

### The *epiA* and *gdmA* deletion mutants have a growth advantage over the parental strains

**Figure 2:**
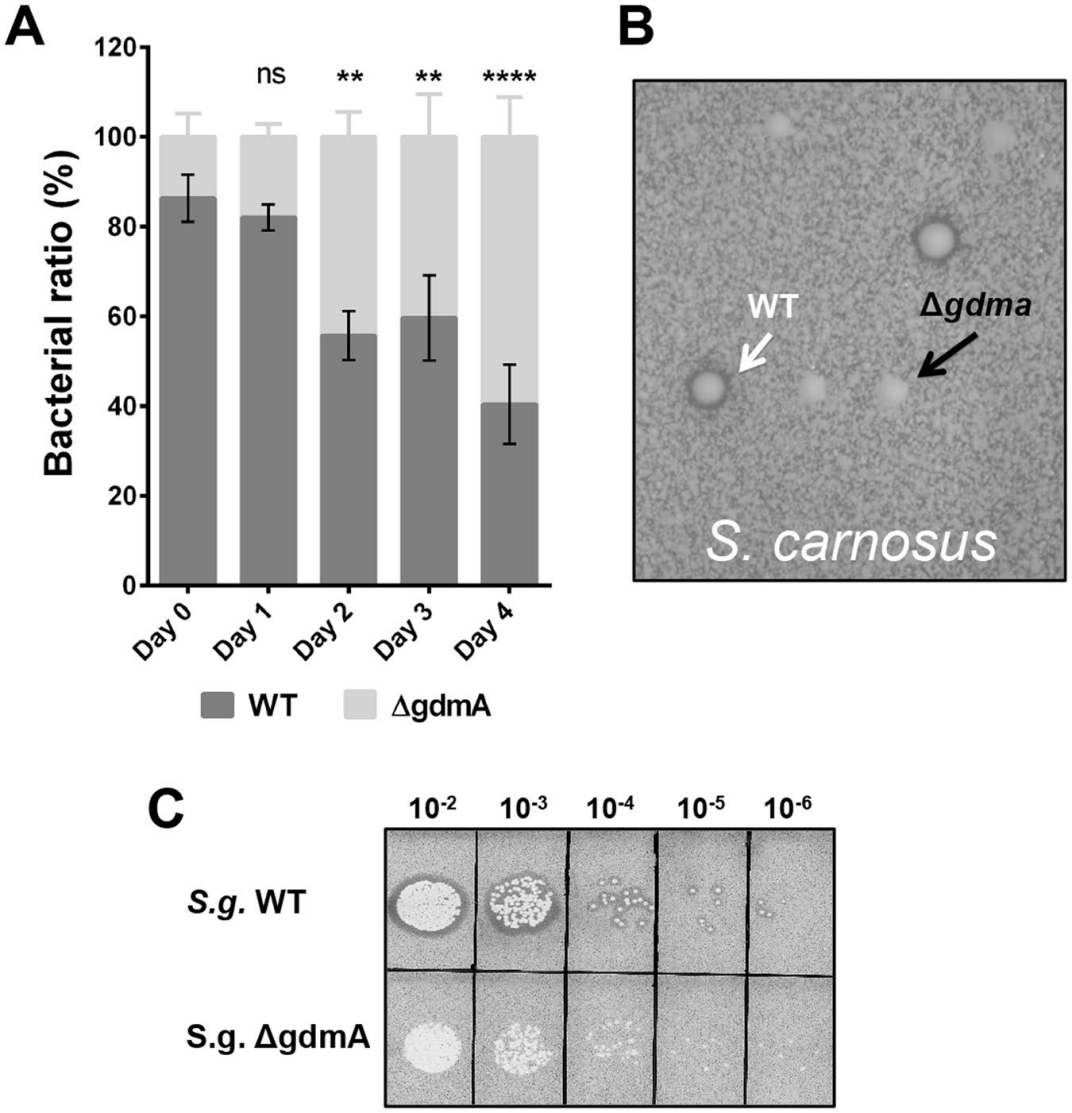
A Gdm deficient mutant can outcompete its parent strain. (**A**) The Gdm deficient mutant gdmA can overgrow the parent strain (dark or light grey columns). Representative data from two independent experiments are shown. For all graphs, each data point represents the mean value ± SD **p < 0.01 ****p < 0.0001 by two-way ANOVA with Bonferroni posttest. (**B**) Colonies of the Δ*gdmA* mutant (white arrow) and the wildtype (black arrow) grown on an agar plate containing *S. carnosus* as biomarker. (**C**) CFU comparison of the *S. gallinarum* parent strain and its *gdmA* mutant on TSA agar containing *S. carnosus*.

The Gdm biosynthesis gene cluster is composed of 11 genes encoding immunity (*gdmFEG*), lantibiotic ABC transporter (*gdmHT*), the Gdm structural gene (*gdmA*), modification genes (*gdm-BCD*), the regulator (*gdmQ*) and the proteolytic processing of pro-Gdm by GdmP^4^. The Epi biosynthesis cluster is similarly organized. We only deleted the structural gene *gdmA* or *epiA* while leaving all the other genes intact. It is worth noting that in the Δ*epiA* and Δ*gdmA* mutants, the immunity genes (*epiFEG* and *gdmFEG*) involved in self-protection are still intact. We also addressed the question whether the mutant can overgrow the parent strains with time. In this regard, we performed a competition assay in which at time zero the parental strain and the mutant were mixed in a ratio of 10:1 OD. The CFU was followed over four days with daily sub culturing in fresh medium. Indeed, the mutant was able to outcompete the parental strain over time (Fig. 2A). After four days the CFU of the mutant increased from 10 to >60% of the population, indicating that the mutant had a clear growth advantage over the parental strain. To distinguish between parental and mutant, diluted cell culture was plated on agar plates containing *S. carnosus* TM300 as test strain because this strain is highly sensitive against Gdm. Gdm-producing parental strain is indicated by colonies with an inhibition zone, while colonies of the mutant did not show such an inhibition zone (Fig. 2B). As a control, serial dilutions were plated on TSA plates containing *S. carnosus* to ensure that it does not cause growth inhibition of either the parental or mutant strains. As expected, both strains grew equally well on the biomarker plates (Fig. 2C).

### The lantibiotic-producing parental strains released more lipids and proteins

**Figure 3:**
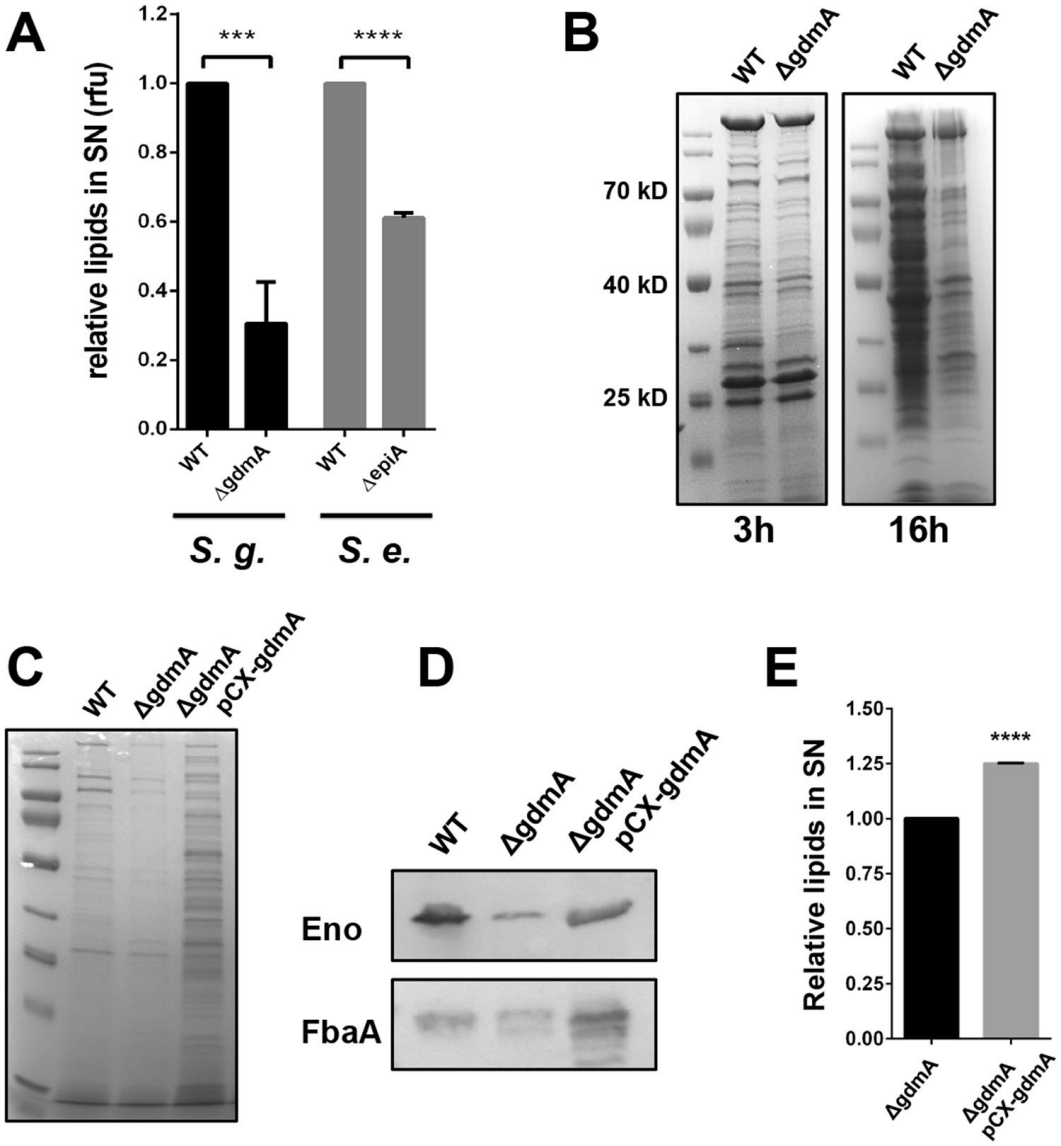
Lantibiotic production causes increased lipid and protein release. (**A**) Relative amount of lipids in *S. gallinarum* and *S. epidermidis* parent strains and their lantibiotic-deficient mutants. Amounts of the mutants are normalized to the corresponding parent strain value. Representative data from three independent experiments are shown, each data point is the mean value ± SD ***p < 0.001 ****p < 0.0001 by students t-test. (**B**) SDS-PAGE (Coomassie blue staining) of extracellular proteins of *S. gallinarum* parent strain and *gdmA* mutant after 3 h and 16 h of growth. (Uncropped gels are shown in Fig. S1). (**C**) Comparison of the overall protein amount of *S. gallinarum* parent strain, the *gdmA* mutant and the complementation pCX-*gdmA*. (**D**) Western blotting for FbaA Eno and GAPDH in the supernatant of *S. gallinarum* parent strain, the gdmA mutant and the complementation pCX-*gdmA*. (**E**) Relative extracellular lipids of the *S. gallinarum gdmA* mutant and the complementation pCX-*gdmA*.

As the two lantibiotics inhibit cell wall biosynthesis by interacting with lipid II, we asked the question whether the integrity of the cytoplasmic membrane is also affected. Thus we compared the release of lipids and proteins in the parental strains and the corresponding *gdmA* and *epiA* mutants. Indeed, in both parent strains the release of membrane-derived lipids was significantly increased compared to the mutant strains (Fig. 3A). *S. gallinarum* released 70% and *S. epidermidis* 40% more lipids than the corresponding mutants. We also investigated the protein release in *S. gallinarum* and found that the parental strain released more proteins into the supernatant than the *gdmA* mutant. The release of proteins in the parental strain was significantly increased with time (Fig. 3B). In addition, both phenotypes of protein and lipid release could be complemented. If *gdmA* was overexpressed in the gdmA mutant (pPCX-*gdmA*) the amount of excreted proteins was drastically increased (Fig. 3C). The Western blot with enolase (Eno) and fructose-1,6 bisphosphate aldolase (FbaA) revealed that these two CPs were also excreted in higher amounts if *gdmA* is overexpressed (Fig. 3D); furthermore, the extracellular lipid content was increased (Fig. 3E).

### Lantibiotic production increased ECP

**Figure 4:**
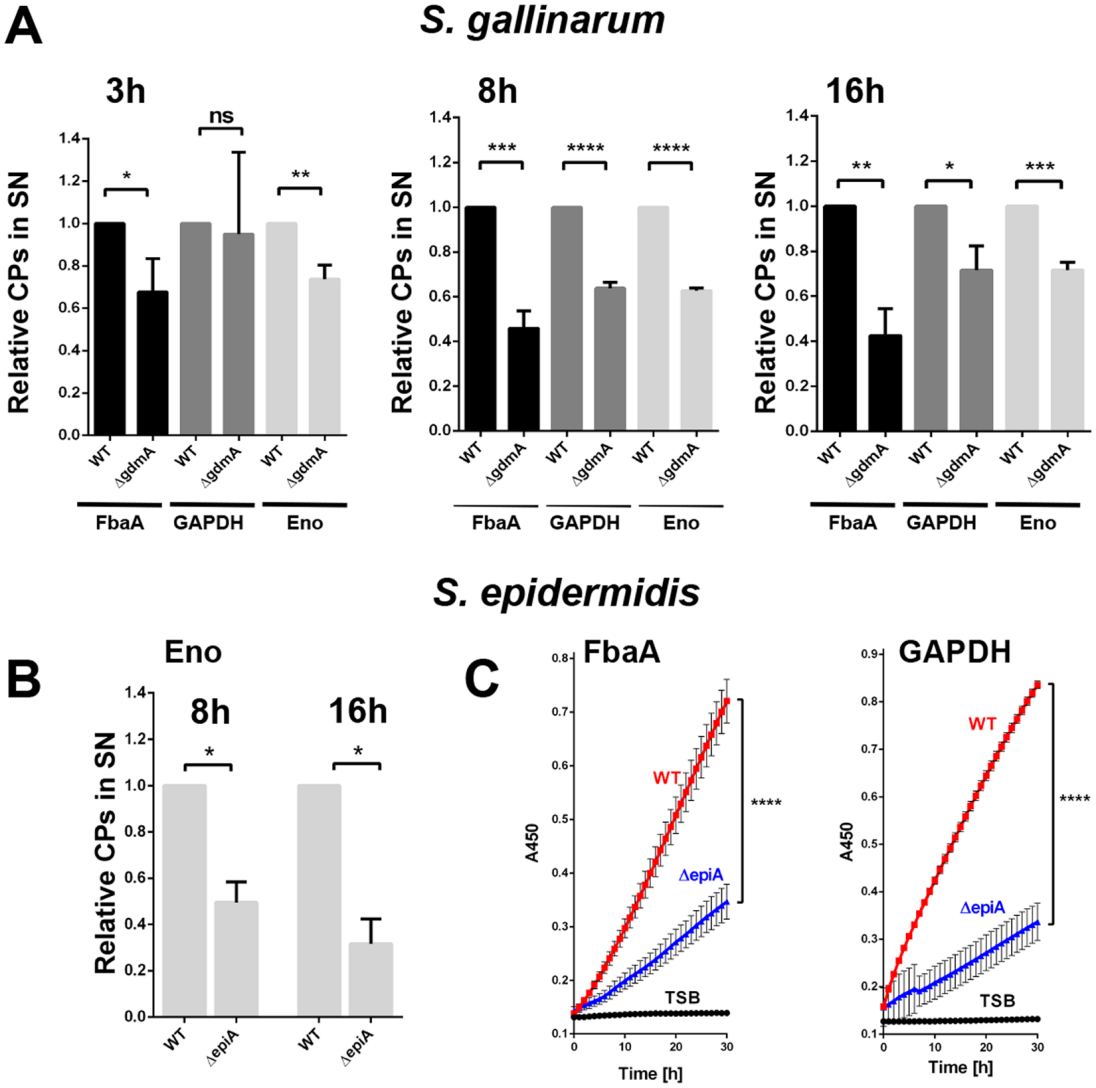
Lantibiotic-deficient mutants release less cytoplasmic proteins compared to the parent strains. (**A**) Relative amounts of FbaA, GAPDH and Eno in the supernatant of *S. gallinarum* and its *gdmA* deletion mutant after 3, 8 and 16 h. (**B**) Relative amounts of Eno in the supernatant of *S. epidermidis* and its *epiA* deletion mutant after 8 and 16 h. FbaA and GAPDH were not detectable. Note: Protein amounts were normalized to that of the parent strain for every protein at any time point tested. Representative data from at least two independent experiments are shown, each data point is the mean value ± SD *p < 0.05, **p < 0.01, ***p < 0.001 ****p < 0.0001 by students t-test. (**C**) Enzymatic assay for detection of FbaA (left) and GAPDH (activity) in culture supernatants of 16 h grown cultures of *S. epidermidis* and its *epiA* mutant. Representative data from at least two independent experiments are shown, each data point is the mean value ± SD ****p < 0.0001 by paired t-test.

For monitoring ECP we selected three model proteins: FbaA, glyceraldehyde-3-phosphate dehydrogenase (GAPDH) and Eno. All three proteins are typical cytoplasmic proteins and play a central role in glycolysis. None of these cytoplasmic enzymes possess a signal peptide for export. We analyzed their release into the supernatant in the parental strains and the corresponding *gdmA* and *epiA* mutants. In *S. gallinarum* parental strain all three cytoplasmic enzymes were released significantly more after 3, 8, and 16 h cultivation than in the *gdmA* mutant (Fig. 4A). In *S. epidermidis*, the same tendency was observed with enolase (Eno) (Fig. 4B). However, we could not detect FbaA and GAPDH in *S. epidermidis*. As the antibodies were made against the *S. aureus* specific FbaA and GAPDH, we assume that the antibodies did not bind efficiently to the *S. epidermidis* counterparts. To confirm our results, we determined the enzymatic activity of FbaA and GAPDH in the supernatant of *S. epidermidis* parental and its *epiA* mutant. In the mutant both activities were decreased compared to the parental strain (Fig. 4C).

### External application of Gdm increased the release of proteins, ATP and lipids

**Figure 5:**
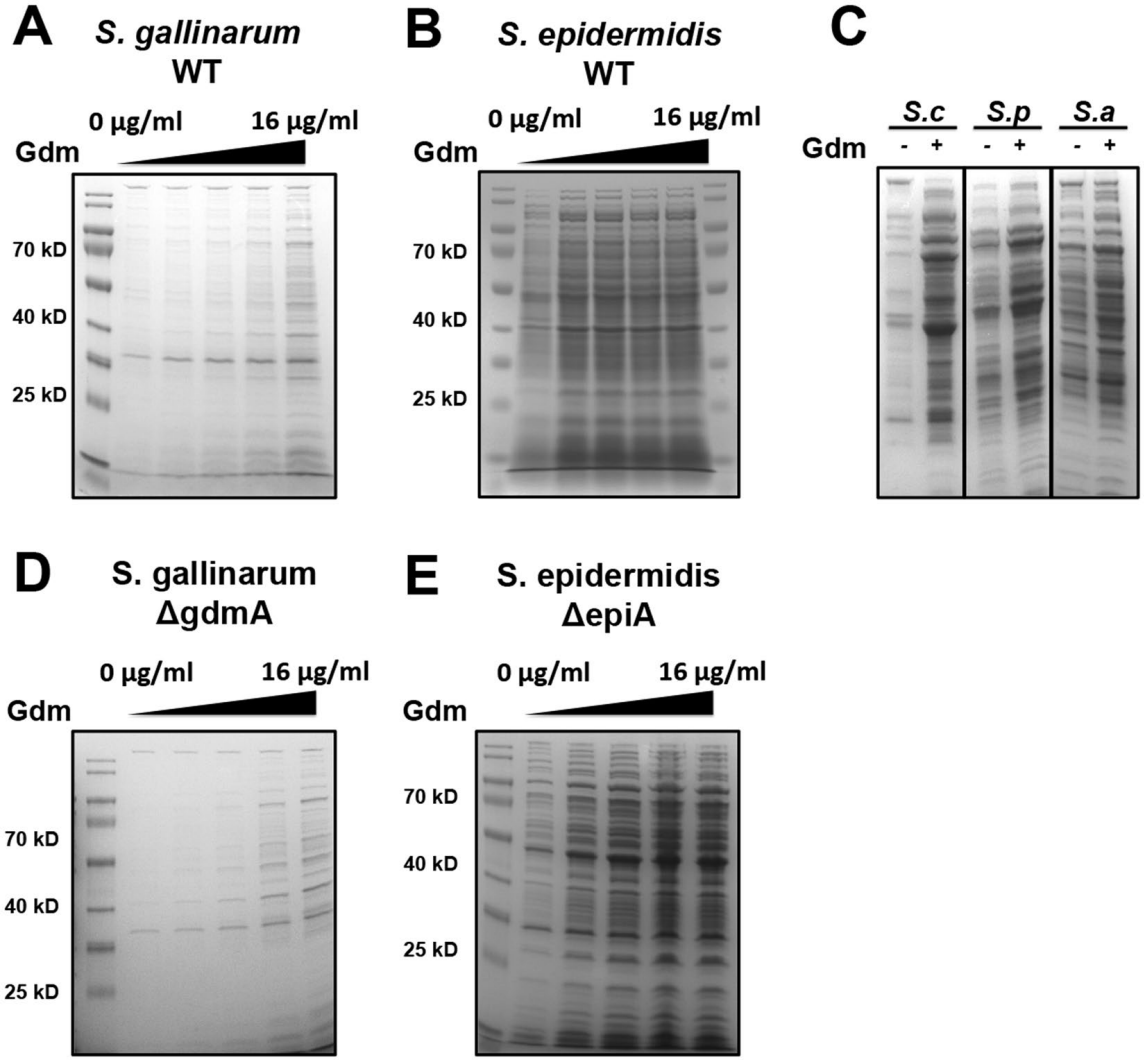
Application of exogenous Gdm increased release of proteins. (**A**) Extracellular proteins of *S. gallinarum* untreated (line 1) and treated with increasing concentrations (2, 4, 8 and 16 μg/ml) of Gdm. (**B**) Extracellular proteins of *S. epidermidis* untreated (line 1) and treated with increasing concentrations (2, 4, 8 and 16 μg/ml) of Gdm. (**C**) Influence 8 μg/ml of Gdm on protein release of the non-lantibiotic producers *S. carnosus*, *S. pseudintermedius* and *S. aureus*. (Uncropped gels are shown in Fig. S1). (**D**) Extracellular proteins of *S. gallinarum gdmA* mutant untreated (line 1) and treated with increasing concentrations (2, 4, 8 and 16 μg/ml) of Gdm. (**E**) Extracellular proteins of *S. epidermidis epiA* mutant untreated (line 1) and treated with increasing concentrations (2, 4, 8 and 16 μg/ml) of Gdm.

Following the observation that endogenous expression of Gdm and Epi increased ECP in the parental strains, we investigated whether exogenous applied lantibiotics have a similar effect. For that, *S. gallinarum* and *S. epidermidis* were cultivated to mid-log phase and after washing (removal of produced lantibiotics) the cells were treated with different concentrations of Gdm (2, 4, 8, 16 μg/ml) for 3 h and the released proteins were visualized on SDS-PAGE. Untreated cells were used as control. In *S. gallinarum* the amount of proteins in the supernatant increased gradually with increasing concentration of Gdm (Fig. 5A). *S. epidermidis* cells were much more susceptible; a clear increase in protein release was observed even at a relatively low concentration of Gdm (2 μg/ml) (Fig. 5B). As control, we also treated non-lantibiotic producing strains such as *S. carnosus* TM300, *S. pseudintermedius* ED99 and *S. aureus* USA300 with Gdm. In all these strains Gdm at 8 μg/ml led to an increased release of proteins (Fig. 5C). Both the *gdmA* and the *epiA* mutant showed a similar effect on exogenous Gdm as their parental strains (Fig. 5D,E).

**Figure 6:**
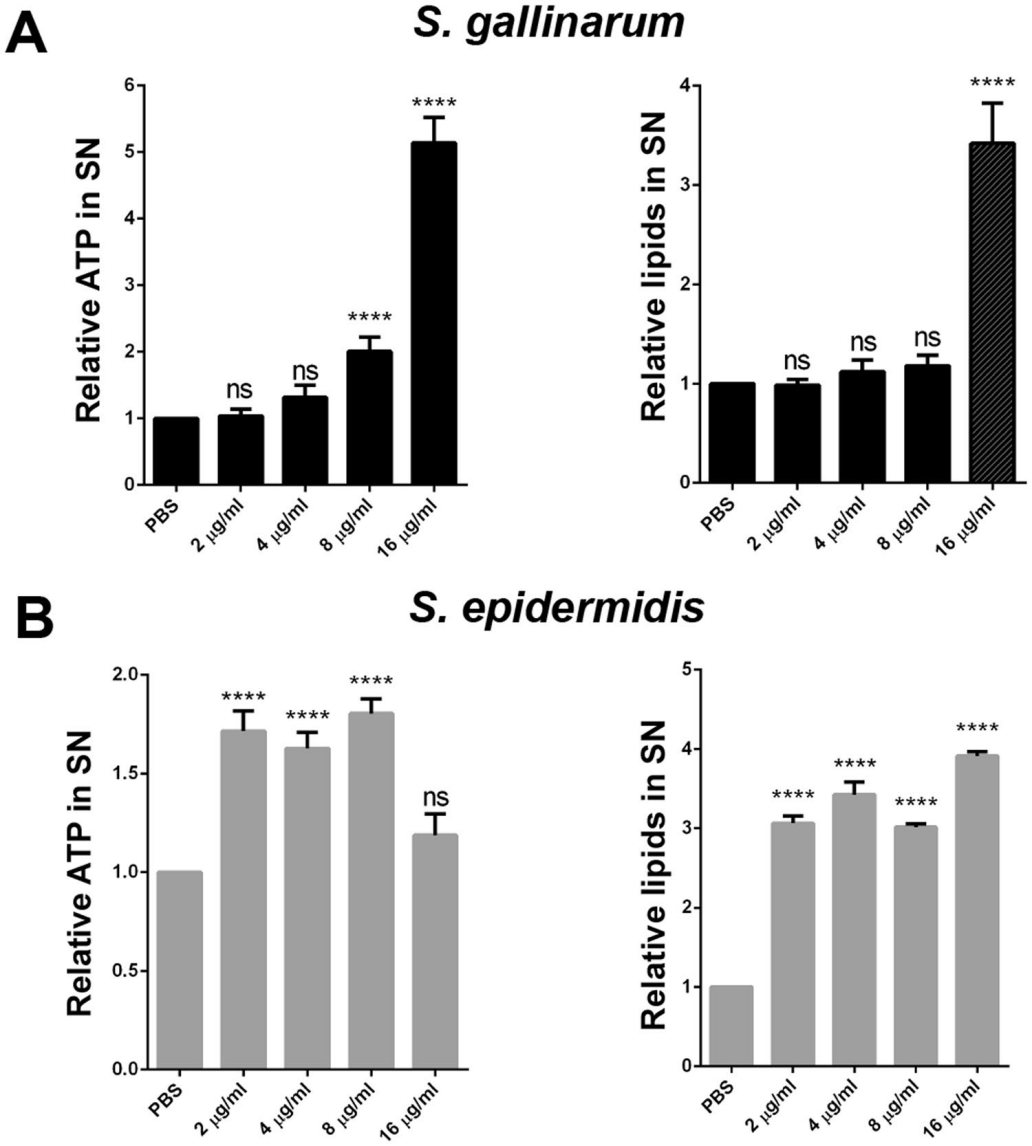
Gdm induced release of ATP and lipids. ATP and lipids were analyzed in the culture supernatants of untreated (PBS) and Gdm treated samples (2, 4, 8, 16 μg/ml) in *S. gallinarum* (**A**) and *S. epidermidis* (**B**). Amounts of ATP and lipids in treated samples were normalized to the untreated control. Representative data from at least two independent experiments are shown, each data point is the mean value ± SD *p < 0.05, **p < 0.01, ***p < 0.001 ****p < 0.0001 ordinary-one-way-ANOVA with Bonferroni post-test.

We assumed that the release of cytoplasmic proteins is due to the disruption of cytoplasmic membrane. Signs of membrane damage are the release of small molecules such as ATP and lipids. Indeed, when *S. gallinarum* and *S. epidermidis* were exposed to Gdm the release of both ATP and lipids into the supernatant was increased (Fig. 6). In *S. gallinarum* higher concentrations (16 μg/ml) of Gdm were necessary (Fig. 6A) than in *S. epidermidis* (2 μg/ml) (Fig. 6B).

## Discussion

In the work described here, we show that the production of lantibiotics Gdm in *S. gallinarum* Tü3928 and Epi in *S. epidermidis* Tü3298 impose a burden on the cells. The production is accompanied by an increased release of cytoplasmic proteins (ECP), ATP and lipids, which is indicative of the dysfunction of the cytoplasmic membrane. Such a phenomenon that the bacteria’s own product causes membrane damage has been recently described for the cytolytic PSMα peptides in *S. aureus*^20^. Here we show that the lantibiotics exert a similar effect as PSMα peptides.

The impairment of the membrane integrity by Gdm and Epi is most likely a secondary effect. It has been shown that the primary target of nisin is lipid I and lipid II, the precursors of murein biosynthesis^21^. Therefore, it was postulated that the primary mechanism of nisin is inhibition of the murein biosynthesis. Later it has been found that nisin also causes pore formation in the membrane, which was accompanied by a breakdown of the membrane potential^22–24^. Gdm and Epi also interact with lipid I, II, III (undecaprenol-pyrophosphate-N-acetyl-glucosamine) and IV (undecaprenol-pyrophosphate-N-acetyl-glucosamine-N-acetyl-mannosamine). Thus, they do not only inhibit murein but also wall teichoic acid (WTA) biosynthesis^10,11^. However, in contrast to nisin, Gdm and Epi are too short (only 22 amino acids long) to span the cytoplasmic membrane of staphylococci and therefore do not form pores like nisin^25,26^. There are several possibilities of how Gdm and Epi exert a membrane damaging effect. Besides murein biosynthesis inhibition which has a strong impact on osmotic destabilization of the membrane, Gdm and Epi also inhibit WTA biosynthesis. In this regard, the cell envelope becomes very fragile, as shown by electron microscopy^11,27^. In addition WTA plays a crucial role in the proton-binding capacity of cell walls as well as controls autolysis mainly via the major autolysin AtlA whose activity is known to decline at acidic pH values^28^. Furthermore, it has been shown that in the absence of WTA, in a *tagO* mutant, autolysis is largely increased by uncontrolled Atl activity^27^. Therefore, we assume that the inhibition of WTA biosynthesis, which is accompanied by increased autolysis activity, also indirectly affects the membrane integrity. Finally, membrane damage can also cause by a breakdown of the turgor pressure if the rigidity of the cell wall is disturbed. This has been demonstrated in a staphylococcal *femB* mutant in which the murein penta-glycine bridge is truncated by two glycine residues^29^.

An unexpected finding was the observation that lantibiotic production is a burden for the producer to the extent that in co-cultivation, the *gdmA* mutant overgrows the lantibiotic-producing parental strain with time. Since the *gdmA* mutant carries the same resistance genes as the parental strain, it is likewise resistant against Gdm. The ABC-transporters EpiFEG/GdmFEG as well as EpiHT/GdmHT convey producer immunity in both the parent and mutant strains^14,30^. The mutant has the advantage that it is less exposed to Gdm than the producer cell, which faces a high concentration of Gdm in its vicinity^14^. We assume that these are the reasons why the *gdmA* mutant overgrows the parental strain with time. This burden of antibiotic production may explain the high prevalence of cryptic (mutated) antibiotic biosynthesis gene clusters in many bacteria such as actinobacteria^31^, bifidobacteria^32^ or staphylococci^33^. For example, *Propionibacterium acnes* and *S. epidermidis* live in close proximity on human skin, and both bacterial species can be isolated from normal and acne vulgaris-affected skin sites. *P. acnes* is highly sensitive to Epi^34^, therefore, if Epi would provide a competitive advantage one would expect to isolate more *S. epidermidis* with anti-propionibacterium activity from acne patients than from healthy volunteers. However, there was no such difference observed^33^.

In native environments, like the skin as an example, staphylococcal lantibiotics are probably not that highly produced than in a static culture under optimal conditions. In a static culture particularly, lantibiotics may accumulate so much that the resistance mechanisms are overwhelmed. This would explain why the parental strains and their corresponding *gdmA* and *epiA* mutants grow similarly until mid-log phase; but thereafter the growth of the wild type strains become increasingly affected (Fig. 1A). We also observed that *S. gallinarum* Tü3928 showed a higher tolerance to Gdm than *S. epidermidis* Tü3298 (Fig. 1B). This might be explained by the fact that *S. gallinarum* Tü3928 was selected for increased productivity, which was associated with increased tolerance to Gdm^35^. The improved growth of the *gdmA* and *epiA* mutants in comparison to their parental strains, shows that the Epi and Gdm biosynthesis machinery as such is not really a burden for its producing-cells. Instead, it is the antimicrobial activity of Gdm and Epi that affects the growth by impairing the membrane integrity at higher concentrations. We therefore postulate that microbial isolates from various ecological niches may frequently contain incomplete or mutated antibiotic biosynthesis clusters.

Our findings question the common belief that antibiotic-producing bacteria have an advantage in securing and maintaining a common ecological niche. If this would be the case, then accordingly all microorganisms should be antibiotic producers. However, in natural habitats like the skin comparatively few lantibiotic-producing strains are found. Our findings for lantibiotics applies most likely also for other antibiotic producing strains where self-protection systems are necessary to comply with the own product, such as the vancomycin producing streptomycetes^17^. In nature, there must be a sophisticated process at work that balances self-protection and self-destruction. Maintaining antibiotic synthesis affords a permanent selective advantage. If this selection pressure is lost, antibiotic production is a burden and gets easily lost - the antibiotic producing industry is fighting permanently with this problem. Empirical medium optimization can overcome such problems to certain extent^36–40^.

## Conclusion

**Figure 7:**
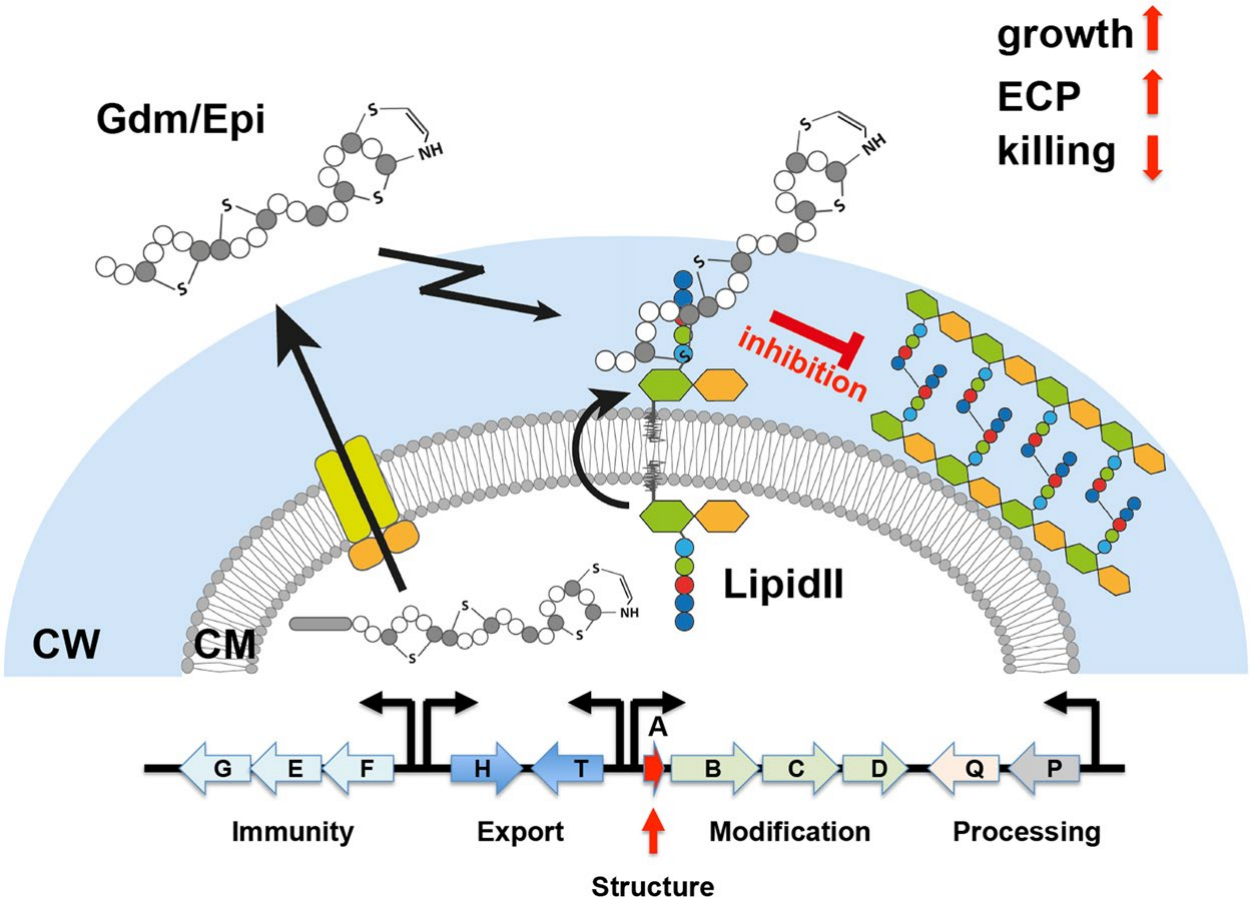
Overview on Gdm and Epis mode of action on the producers. Gallidermin/Epidermin are expressed in an inactive pro-form and secreted by the ABC-tranporter GdmHT/EpiHT, the secreted pro-form is processed by specific protease GdmP/EpiP to active Gdm/Epi. Despite the immunity system Gdm/Epi can inhibit the growth of the producer strain causing enhanced excretion of cytoplasmic proteins and the release of ATP and lipids.

In this study, we show that the production and abundance of the two lantibiotics Epi and Gdm is a burden for its producing-strains. The expression of the lantibiotics in the producer affected growth and caused membrane damage most likely due to the inhibition of cell wall biosynthesis as well as the accompanying osmotic stress. Therefore, it is not surprising that mutants in the structural gene have a growth advantage and are outcompeting the parental strain in a co-cultivation assay. These non-producers, but resistant variants, are in principle ‘cheaters’. They benefit from antibiotic producing neighbors by being protected from competitors but are not burdened with the disadvantage of producing the antibiotic themselves. This is in correlation with the observation that many *Actinomycetes* isolated from soil are multiple antibiotic resistant without producing the antibiotics themselves. This study illustrates how such a situation can arise. A summary graphic of our findings is shown in Fig. 7.

## Material and Methods

### Bacterial strains and growth conditions

The strains used in this study are listed in Table 1. Most studies were carried out with *S. gallinarum* Tü3928 and *S. epidermidis* Tü3298. All strains were cultivated at 37 °C with shaking in basic medium (BM).

**Table 1:** Bacterial strains used in this study.

Strain	Description	Reference
*S. gallinarum* Tü3928	Gallidermin producing strain	^48^
*S. gallinarum* Tü3928 ΔgdmA	Replacement of gdmA by erm^R^	This study
*S. epidermidis* Tü3298	Epidermin producing strain	^1^
*S. epidermidis* Tü3298 ΔepiA	Replacement of epiA by erm^R^	This study
*S. pseudintermedius* ED99		^49^
*S. carnosus* TM300		^50^
*S. aureus* USA300 LAC	CA-MRSA	^51^
*S. gallinarum* pPCX-*gdmA*	Xylose inducible expression of gdmA	This study
*S. gallinarum* pRB473-*gdmFEG*	expression of *gdmFEG* under control of the native promoter	This study

### Allelic replacement

For the construction of a *gdmA* knock-out mutant vector pBK11 was used, which is a temperature-sensitive low-copy *E. coli*-*Staphylococcus* shuttle vector based on fragments from pACYC177 and pTV1ts^41^ including a multiple cloning site. To achieve homologous recombination in the Gdm producer, unusually long flanking regions of *gdmA*, which were about 2 kb in length, were amplified with primer-pairs gdmA F1 down/gdmA F1 upm (TCA**GCTAGC**TTTTAGGGTCTGGATTAATAG/ATT**CTGCAG**TTAACTAGCAATTCGTGG) and gdmA F2 down/gdmA F2 up (TAA**GGATCC**ATGTACTCCTGGATGTGCC/CGT**GAGCTC**TCAGATTTGTTAATATAACTC). After digestion of flanking region F1 with NheI-PstI and flanking region F2 with SacI (treated with Klenow enzyme)-BamHI the fragments were ligated into NheI-Bst1107I restricted pBK11 together with the BamHI-PstI digested erythromycin resistance cassette from pEC2^42^. The final construct pBK11′-inak *gdmA* was electroporated into *S. aureus* RN4220 and after isolation from that cloning host transferred into *S. gallinarum* Tü3928. Homologous recombination was performed as previously described^42^ and *gdmA*-negative colonies were confirmed via PCR and absent Gdm production. For deletion of *epiA*, the plasmid pBB’ermY was used. For construction of pBB’ermY, the plasmid pMcepiDel was digested with StuI. The erythromycin-resistance cassette was digested with HpaI und SmaI from pEC2. Both fragments were ligated, resulting in the plasmid pMcerm. For construction of pBB’ermY, pMcerm was digested with EcoRI and SalI. The resulting fragment was then ligated into pBT6 digested with EcoRI und SalI. This cloning resulted then in pBB’ermY. A schematic overview for the gene replacements are shown in Fig. S2.

### Construction of pPCX-gdmA

For construction of the complementation plasmid pPCX-gdmA, the plasmid pPTX-gdmA^43^ was amplified using “pPT backbone fwd” ACAGGTTTAAGCCTCGCA and “pPT backbone rev” ATTAATATGTACACTATTTCCAAAATTTAAATTCATG. This resulted in a linear fragment containing gdmA and the backbone of pPTX excluding the TetR cassette. For selection, the cat gene from pCtuf-gfp ^44^ was amplified using “Cat fwd” CGAGGCTTAAACCTGTTGCATAATTCAACAGGGTAG and “Cat rev” GAAATAGTGTACATATTAATTAAAGCACCCATTAGTTCAAC. Both fragments were then ligated using HiFi-DNA Assembly Master mix (Thermo Scientific).

### Preparation of biomarker plates

From an overnight culture, 50 ml BM was inoculated to an OD578 = 0.1, cells were grown to an OD578 = 0.6 and diluted to an OD578 = 0.2 with BM. 200 μl were mixed with 3.5 ml BM soft agar and plated on BM agar plates (25 ml BM per plate).

### Bacterial growth competition assay

For the growth competition assay 5 ml TSB medium were inoculated in a ration of 1:10 to a total OD of 0.1, meaning OD = 0.09 of *S. gallinarum* Tü3928 and OD = 0.01 of the Δ*gdmA* mutant and grown for 24 h. After 24 h the resulting culture was re-inoculated in 5 ml fresh TSB medium in a ration of 1:1000. This was repeated until day 4. Every 24 h, the cultures were serially diluted to 10^−8^, subsequently 100 μl of the dilutions 10^−7^ and 10^−8^ were spreaded on TSA agar plates containing *S. carnosus* as biomarker for Gdm production. The agar plates were then incubated for 24 h and colonies containing an inhibition zone versus colonies containing no inhibition zone were counted and the bacterial ratio between parent strain and gdmA mutant was determined. As control we plated serial dilutions of the parent strain and the *gdmA* mutant on the same agar plates. Therefore, mid-log phase cells were diluted to OD_578_ = 0.1, then serial dilutions (10^−1^–10^−6^) were prepared and 10 μl of each dilution were dropped on TSA plates overlaid with *S. carnosus* soft agar.

### Preparation of protein samples for Western blot analysis

Preparation of proteins for Western blot analyses was performed as described in^45,46^. For analysis of proteins in the supernatant cells were inoculated to an OD_578_ = 0.1 and grown for 3, 8 or 16 h and subsequently cells were adjusted to the same OD. For comparison of different deletion mutants, 5 ml at OD_578_ = 10 were collected and harvested by centrifugation at 12000 rpm for 10 min. The supernatant was then mixed with 15 μl of StrataClean resin (Agilent) and incubated under rotation for 15 min at room temperature. The resin was then collected by centrifugation at 12000 rpm for 2 min and washed once in PBS. Subsequently, the resin-pellet was resuspended in 3x Lämmli buffer and boiled for 7 min. 12% Tris-glycin gels (SERVA) were used for all SDS-PAGE experiments. Western blot analysis and quantification was performed as described in^20,45^.

### Relative quantification of cytoplasmic proteins

Photos of Western blots were taken by the gel documentation system (Quantum ST5). As internal standard a parent strain sample was used for each separate Western blot to ensure comparability. Additionally, either by dilution or increasing the volume, it was ensured that the same OD/volume ratio was used for every strain tested in these experiments. The AUC (area under the curve) was determined using ImageJ software. The value of the parent strain was set to 1 and the relative amount of FbaA of each corresponding mutant was calculated using the internal parent strain value. For detection of the CPs, specific rabbit α-FbaA, α-GAPDH and α-Enolase were used as primary antibodies, as secondary antibody a goat-α-rabbit coupled with alkaline phosphatase (Sigma) was used.

### Enzymatic assay for FbaA and GAPDH activity in the supernatant

For detection of FbaA and GAPDH activity in the supernatant. Cultures grown for 16 h were diluted to the same OD (OD_578_ = 9). Then, 1 ml was sterile filtered and 50 μl were used for the assay. For detection the Kits were Aldolase Activity Assay Kit (Colorimetric) and Glyceraldehyde-3-Phosphate Dehydrogenase Activity Assay Kit (Colorimetric) (Abcam) were used. Both assays were used as described by the manufacturer.

### Detection of extracellular membrane lipids and ATP

For lipid analysis, of the parent strain strains and their corresponding deletion mutants, overnight cultures were diluted to the same OD and supernatants were sterile filtered. For investigating the influence of increasing concentrations of Gdm, mid-log phase cells of the strains were washed and equal amounts (1 ml OD_578_ = 2) were resuspended in sterile PBS. Then Gdm (2, 4, 8, 16 μg/ml) was added and incubated for 3 h at 37 °C. Subsequently, the cultures were sterile filtered and used for lipid and ATP detection. Lipid analysis was adapted from^47^. Briefly, membrane lipids were detected and quantified using FM-5-95 (Thermo Fischer). Supernatant samples (100 μl) were mixed with FM-5-95 to a final concentration of 5 μg/ml, and fluorescence was measured with a Tecan microplate reader using excitation at 565 nm and emission at 660 nm. Extracellular ATP levels of resting cell experiments were measured using a BacTiter Glo kit (Promega) according to the manufacturer’s instructions. Background ATP was blanked using sterile PBS.

### Statistical significance

Multiple comparisons were analyzed using one-way or two-way ANOVA with Bonferroni posttest. Normal distributions were analyzed by Student’s t-test. Statistical analyses were performed with GraphPad Prism software, with significance defined as p < 0.05. n represents independent biological replicates.

## Electronic supplementary material

Sup. Figure 1 and 2
