# Supplementary material for: Lantibiotic production is a burden for the producing staphylococci
Source: Sci Rep. 2018 May 10;8:7471. doi: 10.1038/s41598-018-25935-2 (PMC5945643; doi:10.1038/s41598-018-25935-2)
Supplement: Supplementary file 1 — Sup. Figure 1 and 2 [file 41598_2018_25935_MOESM1_ESM.pdf]

Lantibiotic production is a burden for the producing staphylococci

Patrick Ebner[1], Sebastian Reichert[1], Arif Luqman[1], Bernhard Krismer[2] Peter Popella[1,2] and Friedrich Götz[1*]

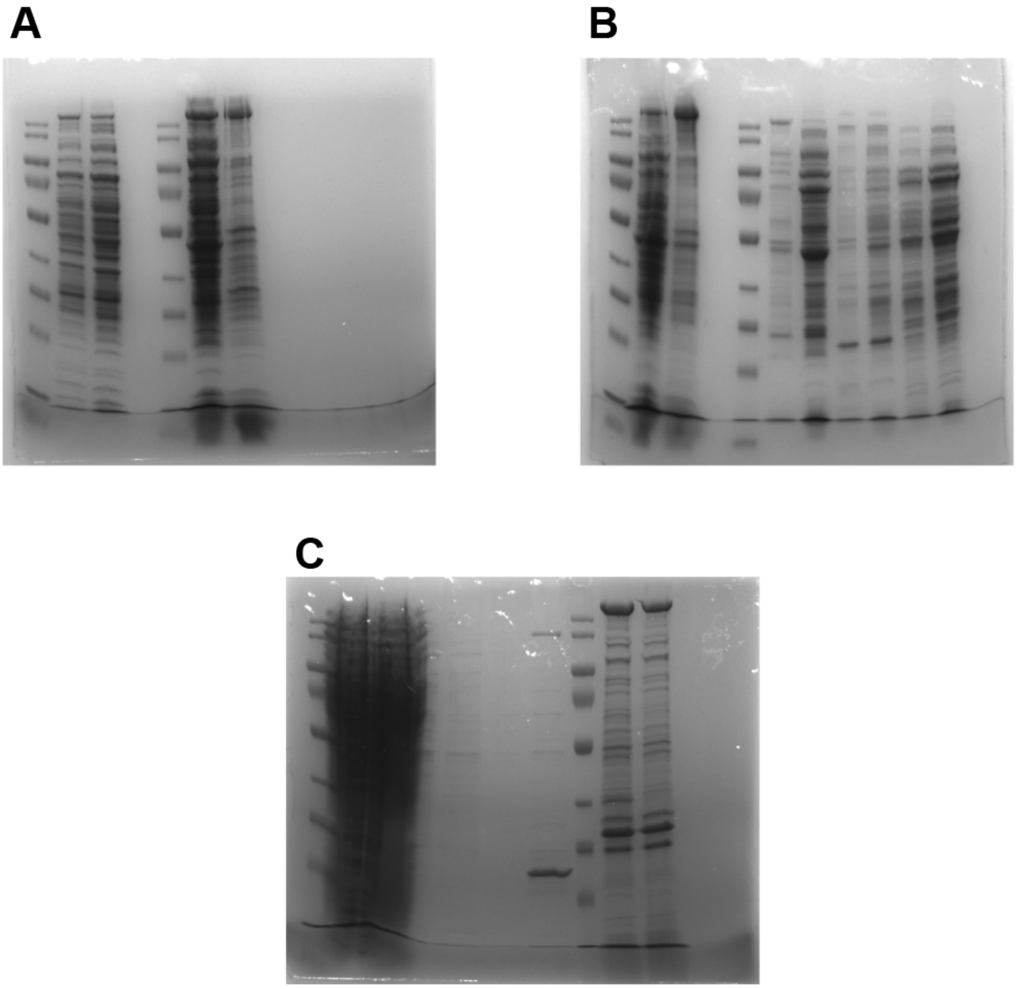

Figure S1: Full SDS-Gels used for Figure preparation. (A) The lanes on the right side were used in main Figure 2B it shows the overall protein amount in the supernatant of *S. gallinarum* WT and ist *gdmA* mutant after 16 h **(B)** The lanes on the right were used for main Figure 4C. the lanes show the proteins in the supernatant of *S. carnosus*, *S. pseudintermidius* and *S. aureus* before and after Gdm treatment **(C)** The lanes on the right were used for main Figure 2A, they show the overall protein amount in the supernatant of *S. gallinarum* WT and ist *gdmA* mutant after 3 h.

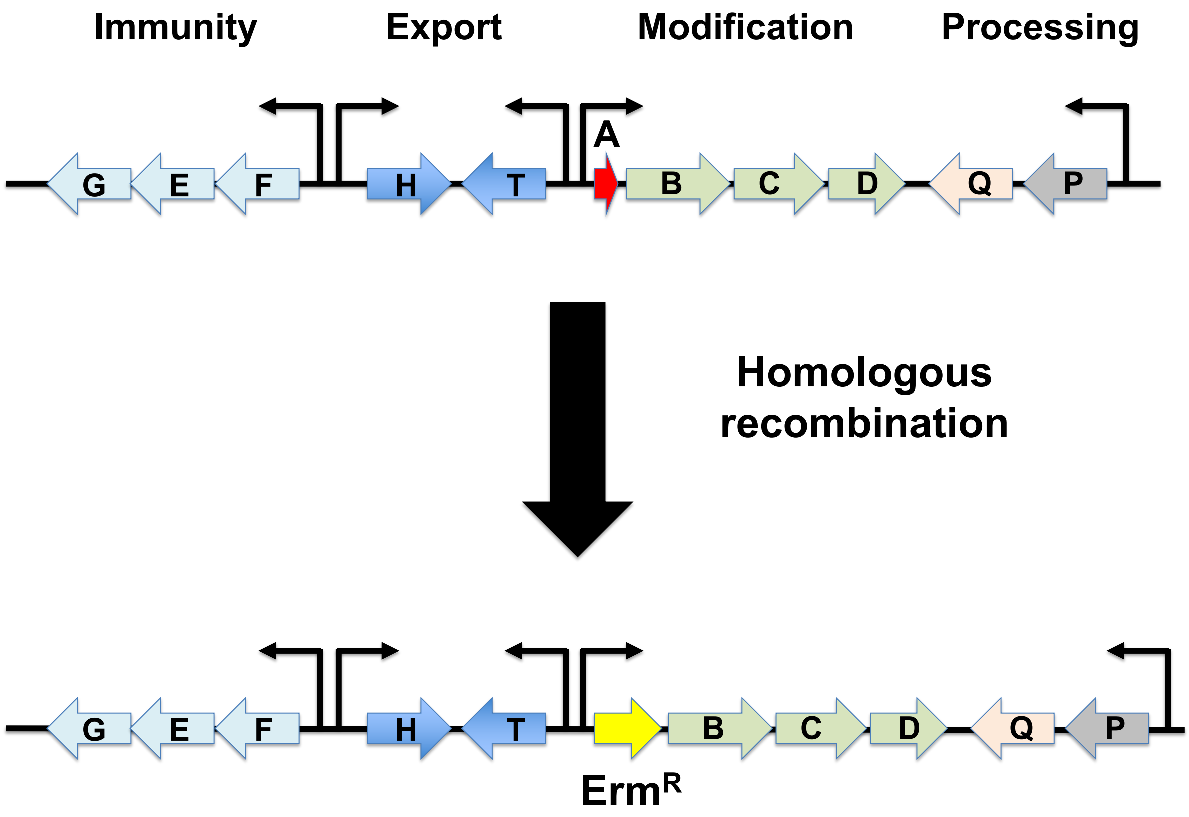

Figure S2: Illustration of the resulting gene-cluster after the allelic replacement experiments. On top, the native gene-cluster harboring all Gdm and Epi biosynthetic genes. On the bottom, the resulting gene-cluster after the allelic replacement, where gdmA or epiA are exchanged by an erythromycin resistance cassette.
